# 2-(2-Carb­oxy­eth­yl)-1,3-dioxoisoindoline-5,6-dicarb­oxy­lic acid methanol monosolvate

**DOI:** 10.1107/S1600536811052755

**Published:** 2011-12-14

**Authors:** Sanaz Khorasani, Manuel A. Fernandes

**Affiliations:** a Molecular Sciences Institute, School of Chemistry, University of the Witwatersrand, PO Wits 2050, Johannesburg, South Africa

## Abstract

In the title compound, C_13_H_9_NO_8_·CH_3_OH, the main mol­ecule possesses three carb­oxy­lic acid groups, which are asymmetrically distributed around the mol­ecule core. This results in hydrogen-bonding motifs ranging from a chain to various rings. The combination of the chain motif together with a carb­oxy­lic dimer *R*_2_^2^(8) ring motif creates a ribbon of mol­ecules propagating along the *c*-axis direction. A second ribbon results from the combination of the chain motif together with a methanol solvent mol­ecule and carboxyl-containing *R*_4_^4^(12) ring motif. These two ribbons combine alternately, forming a hydrogen-bonded layer of mol­ecules parallel to (2
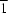
0).

## Related literature

For applications of charge-transfer complexes composed of pyromellitic anhydrides or their imides or polyimide derivatives, see: Barooah *et al.* (2006 ▶); Kim *et al.* (2002 ▶); O’Brien *et al.* (1988 ▶); Dingemans *et al.* (2004 ▶); Zheng *et al.* (2008 ▶). For an example of another asymmetrically substituted diimide, see: Zhu *et al.* (2010 ▶). For a description of the Cambridge Structural Database, see: Allen (2002 ▶). For the *REAXYS* database, see: Elsevier (2011 ▶). For graph-set analysis of hydrogen bonds, see: Etter *et al.* (1990 ▶); Bernstein *et al.* (1995 ▶).
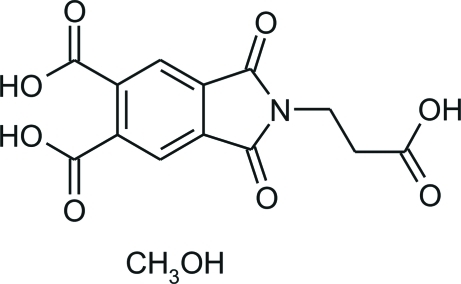


## Experimental

### 

#### Crystal data

C_13_H_9_NO_8_·CH_4_O
*M* *_r_* = 339.25
Triclinic, 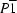

*a* = 8.7830 (3) Å
*b* = 9.7262 (3) Å
*c* = 9.9157 (3) Å
α = 66.164 (2)°
β = 72.830 (2)°
γ = 77.926 (2)°
*V* = 736.35 (4) Å^3^
*Z* = 2
Mo *K*α radiation
μ = 0.13 mm^−1^
*T* = 173 K
0.44 × 0.14 × 0.07 mm

#### Data collection

Bruker APEXII CCD diffractometer
11822 measured reflections
3549 independent reflections
2193 reflections with *I* > 2σ(*I*)
*R*_int_ = 0.053

#### Refinement

*R*[*F* ^2^ > 2σ(*F* ^2^)] = 0.042
*wR*(*F* ^2^) = 0.093
*S* = 0.89
3549 reflections
222 parameters
H-atom parameters constrained
Δρ_max_ = 0.30 e Å^−3^
Δρ_min_ = −0.23 e Å^−3^

### 

Data collection: *APEX2* (Bruker, 2005 ▶); cell refinement: *SAINT* (Bruker, 2005 ▶); data reduction: *SAINT*; program(s) used to solve structure: *SHELXS97* (Sheldrick, 2008 ▶); program(s) used to refine structure: *SHELXL97* (Sheldrick, 2008 ▶); molecular graphics: *ORTEP-3* (Farrugia, 1997 ▶) and *SCHAKAL99* (Keller, 1999 ▶); software used to prepare material for publication: *WinGX* (Farrugia, 1999 ▶) and *PLATON* (Spek, 2009 ▶).

## Supplementary Material

Crystal structure: contains datablock(s) global, I. DOI: 10.1107/S1600536811052755/bh2403sup1.cif

Structure factors: contains datablock(s) I. DOI: 10.1107/S1600536811052755/bh2403Isup2.hkl

Supplementary material file. DOI: 10.1107/S1600536811052755/bh2403Isup3.cml

Additional supplementary materials:  crystallographic information; 3D view; checkCIF report

## Figures and Tables

**Table 1:** Hydrogen-bond geometry (Å, °)

*D*—H⋯*A*	*D*—H	H⋯*A*	*D*⋯*A*	*D*—H⋯*A*
O1—H1⋯O2^i^	0.84	1.84	2.6784 (17)	172
O5—H5⋯O4^ii^	0.84	1.88	2.7181 (15)	178
O7—H7⋯O9^iii^	0.84	1.71	2.5360 (17)	168
O9—H9⋯O8	0.84	1.87	2.7014 (17)	169

Symmetry codes: (i) 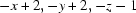; (ii) 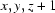; (iii) 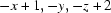.

**Fig. 1.**
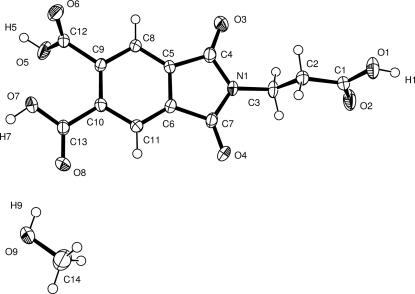
The molecular structure of the title compound, showing displacement ellipsoids at the 50% probability level for non-H atoms.

**Fig. 2.**
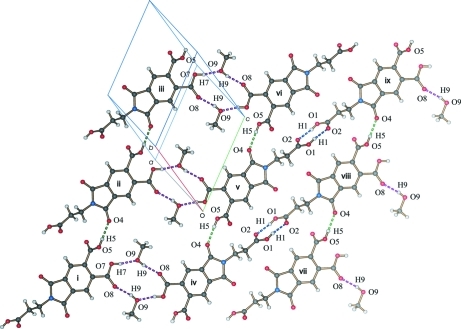
Diagram showing the intermolecular O—H···O hydrogen bonding network in the structure of the title solvate. The molecule is asymmetric and as a consequence forms different hydrogen bonded ribbons on opposite sides of the molecule. The combinations of these results in a H-bonded sheet running parallel to (-2 1 0). Symmetry operators for molecules in diagram: (i) x, y, -2+z; (ii) x, y, -1+z; (iii) x, y, z; (iv) 1-x, -y, -z; (v) 1-x, -y, 1-z; (vi) 1-x, -y, 2-z; (vii) -1+x, -2+y, z; (viii) -1+x, -2+y, 1+z; (ix) -1+x, -2+y, 2+z.

## supplementary crystallographic information

### Comment

Charge transfer complexes composed of pyromellitic anhydride have been extensively studied for their electronic properties. These molecules have also been modified into various imides or polyimides by reaction with suitable amines for use as host guest materials (Barooah *et al.*, 2006), gas separation materials (Kim *et al.*, 2002; O'Brien *et al.*, 1988), and semiconductor materials (Dingemans *et al.*, 2004; Zheng *et al*., 2008). Such products are usually symmetric with very few asymmetric examples of these products having been reported. A search of the *REAXYS* database (Elsevier, 2011) of reactions involving pyromellitic anhydride as starting material resulted in 1083 hits. Of these, very few report asymmetric products and only five reported reactions result in one side of the molecule being converted to an imide while the other side is opened resulting in a di-acid. A search of asymmetric pyromellitic anhydride derived molecules in the Cambridge Structural Database (*CSD*; Version 5.32 release; Allen, 2002) indicates that only one asymmetrically substituted molecule (a diimide) has been reported (Zhu *et al.*, 2010). No structure involving a pyromellitic molecule which has been converted to an imide on one side, and had the other side ring opened to form a di-acid has been reported. Due to asymmetry in carboxylic acid substitution, such a molecule should result in an interesting and unusual hydrogen bonded network.

The title molecule (Fig. 1) has three carboxylic acid groups capable of H-bonding distributed unevenly as two *ortho* to each other on one side of the molecule, and another as a propionic acid extending out from the imide group on the other side of the molecule. This difference in carboxylic acid location results in different hydrogen bond patterns on the opposite sides of the molecule.

The crystal structure is composed of hydrogen bonded layers of molecules which are stacked along the [-2 1 0] direction. Each layer is held together by several hydrogen bonds (Fig. 2). On the imide side, the single propionic acid hydrogen bonds to another on a neighbouring molecule (related by an inversion center) to form a carboxylic acid dimer which can be described by the graph set *R*_2_^2^(8) (Etter *et al.*, 1990; Bernstein *et al.*, 1995). On the other side of the molecule, one of the carboxylic groups hydrogen bonds to a carbonyl group of a neighbouring molecule (related by translation along *c*) to form a chain which can be described by the graph set *C*(9). The combination of the *C*(9) chain and the *R*_2_^2^(8) dimers results in a ring of hydrogen bonded molecules described by the graph set *R*_4_^4^(40). This upon cell translation produces a ribbon of molecules down the *c*-axis. A second different ribbon exists on the edges of the one just described. This is formed by the remaining carboxylic acid group which together with the methanol molecule forms a centrosymmetric hydrogen bonded ring of molecules [graph set *R*_4_^4^(12)] to link the previously mentioned ribbons together. The combination of the two alternating ribbons results in a hydrogen bonded layer of molecules parallel to (2 -1 0).

### Experimental

The title compound was accidently synthesized in a crude yield of 35% by reaction of pyromellitic anhydride with beta-alanine in a 1:1 molar ratio by refluxing in DMF containing water as an impurity. The product from the reaction was recrystallized for analysis by X-ray diffraction from methanol by means of slow evaporation at room temperature resulting in colorless needle-like crystals.

### Refinement

All H atoms were positioned geometrically, and allowed to ride on their parent atoms, with Atom—H bond lengths of 0.95 Å (CH), 0.99 Å (CH_2_), 0.98 Å (CH_3_), or 0.84 Å (OH). Isotropic displacement parameters for these atoms were set to 1.2 times *U*_eq_ of the parent atom for CH and CH_2_, and 1.5 times *U*_eq_ of the parent atom for CH_3_ and OH.

### Crystal data

**Table:**

C_13_H_9_NO_8_·CH_4_O	*Z* = 2
*M**_r_* = 339.25	*F*(000) = 352
Triclinic, *P*1	*D*_x_ = 1.530 Mg m^−^^3^
Hall symbol: -P 1	Mo *K*α radiation, λ = 0.71073 Å
*a* = 8.7830 (3) Å	Cell parameters from 2207 reflections
*b* = 9.7262 (3) Å	θ = 2.3–26.5°
*c* = 9.9157 (3) Å	µ = 0.13 mm^−^^1^
α = 66.164 (2)°	*T* = 173 K
β = 72.830 (2)°	Needle, colourless
γ = 77.926 (2)°	0.44 × 0.14 × 0.07 mm
*V* = 736.35 (4) Å^3^	

### Data collection

**Table:**

Bruker APEXII CCD diffractometer	2193 reflections with *I* > 2σ(*I*)
Radiation source: fine-focus sealed tube	*R*_int_ = 0.053
graphite	θ_max_ = 28.0°, θ_min_ = 2.3°
φ and ω scans	*h* = −11→11
11822 measured reflections	*k* = −12→12
3549 independent reflections	*l* = −13→13

### Refinement

**Table:**

Refinement on *F*^2^	Primary atom site location: structure-invariant direct methods
Least-squares matrix: full	Secondary atom site location: difference Fourier map
*R*[*F*^2^ > 2σ(*F*^2^)] = 0.042	Hydrogen site location: inferred from neighbouring sites
*wR*(*F*^2^) = 0.093	H-atom parameters constrained
*S* = 0.89	*w* = 1/[σ^2^(*F*_o_^2^) + (0.0403*P*)^2^] where *P* = (*F*_o_^2^ + 2*F*_c_^2^)/3
3549 reflections	(Δ/σ)_max_ < 0.001
222 parameters	Δρ_max_ = 0.30 e Å^−^^3^
0 restraints	Δρ_min_ = −0.23 e Å^−^^3^
0 constraints	

### Fractional atomic coordinates and isotropic or equivalent isotropic displacement parameters (Å^2^)

**Table:**

	*x*	*y*	*z*	*U*_iso_*/*U*_eq_	
C1	0.9965 (2)	0.8792 (2)	−0.28914 (19)	0.0275 (4)	
C2	0.9929 (2)	0.79281 (19)	−0.12438 (17)	0.0255 (4)	
H2A	1.0792	0.8206	−0.0977	0.031*	
H2B	1.0119	0.6831	−0.1046	0.031*	
C3	0.8321 (2)	0.8275 (2)	−0.02696 (17)	0.0283 (4)	
H3A	0.8179	0.9358	−0.0413	0.034*	
H3B	0.7459	0.8090	−0.0614	0.034*	
C4	0.8660 (2)	0.7795 (2)	0.23163 (18)	0.0256 (4)	
C5	0.81809 (19)	0.66165 (18)	0.38558 (17)	0.0211 (4)	
C6	0.74964 (19)	0.55402 (19)	0.37121 (18)	0.0217 (4)	
C7	0.74729 (19)	0.60218 (19)	0.20843 (18)	0.0240 (4)	
C8	0.83220 (19)	0.65153 (19)	0.52394 (17)	0.0226 (4)	
H8	0.8806	0.7256	0.5322	0.027*	
C9	0.77322 (19)	0.52920 (19)	0.65146 (17)	0.0208 (4)	
C10	0.70651 (19)	0.41746 (18)	0.63744 (18)	0.0211 (4)	
C11	0.69438 (19)	0.42998 (18)	0.49515 (17)	0.0232 (4)	
H11	0.6494	0.3552	0.4845	0.028*	
C12	0.7872 (2)	0.52609 (19)	0.80077 (18)	0.0234 (4)	
C13	0.6457 (2)	0.28193 (19)	0.77029 (18)	0.0235 (4)	
N1	0.81581 (16)	0.73687 (16)	0.13388 (14)	0.0243 (3)	
O1	1.13876 (15)	0.87456 (16)	−0.38054 (13)	0.0387 (3)	
H1	1.1320	0.9237	−0.4707	0.058*	
O2	0.87748 (15)	0.94623 (17)	−0.33501 (13)	0.0441 (4)	
O3	0.93188 (16)	0.89010 (14)	0.19466 (14)	0.0379 (3)	
O4	0.69235 (14)	0.53863 (14)	0.15292 (12)	0.0312 (3)	
O5	0.64890 (14)	0.55908 (16)	0.88490 (13)	0.0352 (3)	
H5	0.6617	0.5507	0.9686	0.053*	
O6	0.91487 (14)	0.50637 (15)	0.83081 (13)	0.0347 (3)	
O7	0.69234 (15)	0.25746 (15)	0.89223 (13)	0.0371 (3)	
H7	0.6421	0.1897	0.9661	0.056*	
O8	0.56037 (15)	0.20381 (14)	0.76129 (13)	0.0339 (3)	
C14	0.4653 (2)	−0.0897 (2)	0.7258 (2)	0.0445 (5)	
H14A	0.4025	−0.0060	0.6629	0.067*	
H14B	0.4247	−0.1853	0.7493	0.067*	
H14C	0.5780	−0.0921	0.6706	0.067*	
O9	0.45194 (18)	−0.06903 (15)	0.86241 (13)	0.0442 (4)	
H9	0.4789	0.0164	0.8421	0.066*	

### Atomic displacement parameters (Å^2^)

**Table:**

	*U*^11^	*U*^22^	*U*^33^	*U*^12^	*U*^13^	*U*^23^
C1	0.0325 (10)	0.0322 (10)	0.0191 (9)	−0.0140 (9)	−0.0007 (8)	−0.0093 (8)
C2	0.0333 (10)	0.0245 (9)	0.0185 (9)	−0.0081 (8)	−0.0045 (7)	−0.0063 (8)
C3	0.0367 (10)	0.0311 (10)	0.0119 (9)	−0.0064 (8)	−0.0047 (7)	−0.0018 (8)
C4	0.0290 (10)	0.0273 (10)	0.0188 (9)	−0.0038 (8)	−0.0030 (7)	−0.0081 (8)
C5	0.0238 (9)	0.0221 (9)	0.0159 (9)	−0.0066 (7)	−0.0018 (7)	−0.0054 (7)
C6	0.0244 (9)	0.0260 (9)	0.0157 (8)	−0.0041 (7)	−0.0036 (7)	−0.0087 (7)
C7	0.0257 (9)	0.0299 (10)	0.0160 (9)	−0.0036 (8)	−0.0029 (7)	−0.0091 (8)
C8	0.0286 (9)	0.0243 (9)	0.0164 (9)	−0.0097 (8)	−0.0031 (7)	−0.0073 (7)
C9	0.0222 (9)	0.0254 (9)	0.0155 (8)	−0.0037 (7)	−0.0038 (6)	−0.0080 (7)
C10	0.0226 (9)	0.0225 (9)	0.0173 (9)	−0.0048 (7)	−0.0014 (7)	−0.0074 (7)
C11	0.0287 (9)	0.0237 (9)	0.0187 (9)	−0.0087 (8)	−0.0024 (7)	−0.0089 (8)
C12	0.0299 (10)	0.0241 (9)	0.0156 (9)	−0.0081 (8)	−0.0035 (7)	−0.0053 (7)
C13	0.0282 (9)	0.0235 (9)	0.0167 (9)	−0.0043 (8)	−0.0031 (7)	−0.0060 (8)
N1	0.0310 (8)	0.0269 (8)	0.0128 (7)	−0.0074 (7)	−0.0042 (6)	−0.0036 (6)
O1	0.0377 (8)	0.0503 (9)	0.0181 (7)	−0.0034 (6)	−0.0008 (5)	−0.0071 (7)
O2	0.0344 (8)	0.0690 (11)	0.0175 (7)	−0.0089 (7)	−0.0066 (6)	−0.0024 (7)
O3	0.0554 (9)	0.0322 (8)	0.0251 (7)	−0.0218 (7)	−0.0048 (6)	−0.0043 (6)
O4	0.0407 (8)	0.0404 (8)	0.0193 (7)	−0.0116 (6)	−0.0072 (5)	−0.0140 (6)
O5	0.0324 (7)	0.0571 (9)	0.0191 (7)	−0.0022 (6)	−0.0051 (5)	−0.0187 (7)
O6	0.0311 (7)	0.0506 (9)	0.0271 (7)	−0.0064 (6)	−0.0097 (6)	−0.0159 (7)
O7	0.0505 (8)	0.0376 (8)	0.0194 (7)	−0.0229 (7)	−0.0103 (6)	0.0036 (6)
O8	0.0465 (8)	0.0300 (7)	0.0269 (7)	−0.0198 (6)	−0.0057 (6)	−0.0066 (6)
C14	0.0469 (13)	0.0542 (14)	0.0352 (12)	−0.0073 (11)	−0.0103 (9)	−0.0176 (11)
O9	0.0705 (10)	0.0378 (9)	0.0234 (7)	−0.0297 (8)	−0.0035 (7)	−0.0038 (6)

### Geometric parameters (Å, °)

**Table:**

C1—O2	1.217 (2)	C8—H8	0.9500
C1—O1	1.313 (2)	C9—C10	1.407 (2)
C1—C2	1.499 (2)	C9—C12	1.509 (2)
C2—C3	1.517 (2)	C10—C11	1.400 (2)
C2—H2A	0.9900	C10—C13	1.495 (2)
C2—H2B	0.9900	C11—H11	0.9500
C3—N1	1.4570 (19)	C12—O6	1.2013 (19)
C3—H3A	0.9900	C12—O5	1.3153 (19)
C3—H3B	0.9900	C13—O8	1.2159 (18)
C4—O3	1.2014 (19)	C13—O7	1.3062 (19)
C4—N1	1.399 (2)	O1—H1	0.8400
C4—C5	1.493 (2)	O5—H5	0.8400
C5—C8	1.376 (2)	O7—H7	0.8400
C5—C6	1.383 (2)	C14—O9	1.417 (2)
C6—C11	1.377 (2)	C14—H14A	0.9800
C6—C7	1.493 (2)	C14—H14B	0.9800
C7—O4	1.2142 (18)	C14—H14C	0.9800
C7—N1	1.382 (2)	O9—H9	0.8400
C8—C9	1.392 (2)		
			
O2—C1—O1	122.62 (16)	C9—C8—H8	121.1
O2—C1—C2	122.97 (15)	C8—C9—C10	120.64 (14)
O1—C1—C2	114.40 (15)	C8—C9—C12	115.64 (14)
C1—C2—C3	110.35 (14)	C10—C9—C12	123.72 (14)
C1—C2—H2A	109.6	C11—C10—C9	120.38 (14)
C3—C2—H2A	109.6	C11—C10—C13	116.95 (14)
C1—C2—H2B	109.6	C9—C10—C13	122.67 (14)
C3—C2—H2B	109.6	C6—C11—C10	117.92 (14)
H2A—C2—H2B	108.1	C6—C11—H11	121.0
N1—C3—C2	112.98 (14)	C10—C11—H11	121.0
N1—C3—H3A	109.0	O6—C12—O5	125.07 (15)
C2—C3—H3A	109.0	O6—C12—C9	121.79 (15)
N1—C3—H3B	109.0	O5—C12—C9	112.91 (14)
C2—C3—H3B	109.0	O8—C13—O7	124.65 (15)
H3A—C3—H3B	107.8	O8—C13—C10	121.21 (15)
O3—C4—N1	125.36 (16)	O7—C13—C10	114.15 (14)
O3—C4—C5	129.03 (15)	C7—N1—C4	112.18 (13)
N1—C4—C5	105.61 (14)	C7—N1—C3	124.74 (13)
C8—C5—C6	121.89 (14)	C4—N1—C3	123.07 (14)
C8—C5—C4	130.10 (15)	C1—O1—H1	109.5
C6—C5—C4	108.01 (14)	C12—O5—H5	109.5
C11—C6—C5	121.31 (15)	C13—O7—H7	109.5
C11—C6—C7	130.59 (14)	O9—C14—H14A	109.5
C5—C6—C7	108.08 (14)	O9—C14—H14B	109.5
O4—C7—N1	126.69 (15)	H14A—C14—H14B	109.5
O4—C7—C6	127.21 (15)	O9—C14—H14C	109.5
N1—C7—C6	106.07 (13)	H14A—C14—H14C	109.5
C5—C8—C9	117.82 (14)	H14B—C14—H14C	109.5
C5—C8—H8	121.1	C14—O9—H9	109.5
			
O2—C1—C2—C3	−11.2 (2)	C5—C6—C11—C10	1.3 (2)
O1—C1—C2—C3	169.92 (14)	C7—C6—C11—C10	−177.13 (16)
C1—C2—C3—N1	174.94 (14)	C9—C10—C11—C6	0.1 (2)
O3—C4—C5—C8	2.3 (3)	C13—C10—C11—C6	179.96 (14)
N1—C4—C5—C8	−176.91 (16)	C8—C9—C12—O6	−67.8 (2)
O3—C4—C5—C6	−178.39 (18)	C10—C9—C12—O6	111.82 (19)
N1—C4—C5—C6	2.44 (18)	C8—C9—C12—O5	106.95 (17)
C8—C5—C6—C11	−1.0 (2)	C10—C9—C12—O5	−73.4 (2)
C4—C5—C6—C11	179.60 (15)	C11—C10—C13—O8	−14.9 (2)
C8—C5—C6—C7	177.76 (15)	C9—C10—C13—O8	164.96 (16)
C4—C5—C6—C7	−1.66 (18)	C11—C10—C13—O7	165.02 (15)
C11—C6—C7—O4	0.8 (3)	C9—C10—C13—O7	−15.1 (2)
C5—C6—C7—O4	−177.83 (17)	O4—C7—N1—C4	179.47 (17)
C11—C6—C7—N1	178.86 (17)	C6—C7—N1—C4	1.36 (18)
C5—C6—C7—N1	0.27 (18)	O4—C7—N1—C3	0.7 (3)
C6—C5—C8—C9	−0.7 (2)	C6—C7—N1—C3	−177.39 (14)
C4—C5—C8—C9	178.53 (16)	O3—C4—N1—C7	178.45 (17)
C5—C8—C9—C10	2.1 (2)	C5—C4—N1—C7	−2.34 (18)
C5—C8—C9—C12	−178.23 (15)	O3—C4—N1—C3	−2.8 (3)
C8—C9—C10—C11	−1.8 (2)	C5—C4—N1—C3	176.44 (14)
C12—C9—C10—C11	178.56 (16)	C2—C3—N1—C7	−93.99 (19)
C8—C9—C10—C13	178.31 (15)	C2—C3—N1—C4	87.39 (19)
C12—C9—C10—C13	−1.3 (2)		

### Hydrogen-bond geometry (Å, °)

**Table:**

*D*—H···*A*	*D*—H	H···*A*	*D*···*A*	*D*—H···*A*
O1—H1···O2^i^	0.84	1.84	2.6784 (17)	172
O5—H5···O4^ii^	0.84	1.88	2.7181 (15)	178
O7—H7···O9^iii^	0.84	1.71	2.5360 (17)	168
O9—H9···O8	0.84	1.87	2.7014 (17)	169

Symmetry codes: (i) −*x*+2, −*y*+2, −*z*−1; (ii) *x*, *y*, *z*+1; (iii) −*x*+1, −*y*, −*z*+2.
